# TRIM25 ubiquitinates and degrades p62/SQSTM1 to suppress autophagy: TRIM25 ubiquitinates and degrades p62/SQSTM1

**DOI:** 10.3724/abbs.2025062

**Published:** 2025-06-20

**Authors:** Xiang Qiu, Jin Ren, Yun Yang, Weikang Hu, Chengcheng Wang, Ronggui Hu, Chuanyin Li

**Affiliations:** 1 Medical College of Guizhou University Guiyang 550025 China; 2 State Key Laboratory of Molecular Biology Shanghai Institute of Biochemistry and Cell Biology Center for Excellence in Molecular Cell Science Chinese Academy of Sciences Shanghai 200031 China; 3 Key Laboratory of Systems Health Science of Zhejiang Province School of Life Science Hangzhou Institute for Advanced Study University of Chinese Academy of Sciences Hangzhou 310024 China; 4 Department of Colorectal Surgery and Oncology (Key Laboratory of Cancer Prevention and Intervention China National Ministry of Education Key Laboratory of Molecular Biology in Medical Sciences Zhejiang Province China) the Second Affiliated Hospital Zhejiang University School of Medicine Hangzhou 310009 China

Autophagy is a conserved catabolic process in which organelles, macromolecules and pathogens are degraded via lysosomes [ 1, 2]. Sequestosome 1 (SQSTM1), also known as p62, the first autophagy receptor identified, binds to ubiquitin on targets and LC3 on phagophores, mediating the selective autophagy of ubiquitinated substrates [ 3, 4]. To identify the potential interacting partners for p62, Flag-tagged p62 was transfected into HEK293T cells and used as bait to Co-immunoprecipitate (Co-IP) with proteins that form a complex with p62. More materials and methods are provided in the Supplementary Materials and Methods. The proteins were then identified via mass spectrometry analysis. Two E3 ubiquitin ligases, TRIM25 and ITCH, were identified as the highest confidence hits in a list of identified proteins ( Figure 1A and Supplementary Table S1). Gene Ontology (GO) analysis revealed that p62-interacting proteins were enriched in the ubiquitin-dependent protein degradation, protein folding, oxidative phosphorylation, autophagy, *etc*., signalling pathways ( Figure 1B).

**Figure 1:**
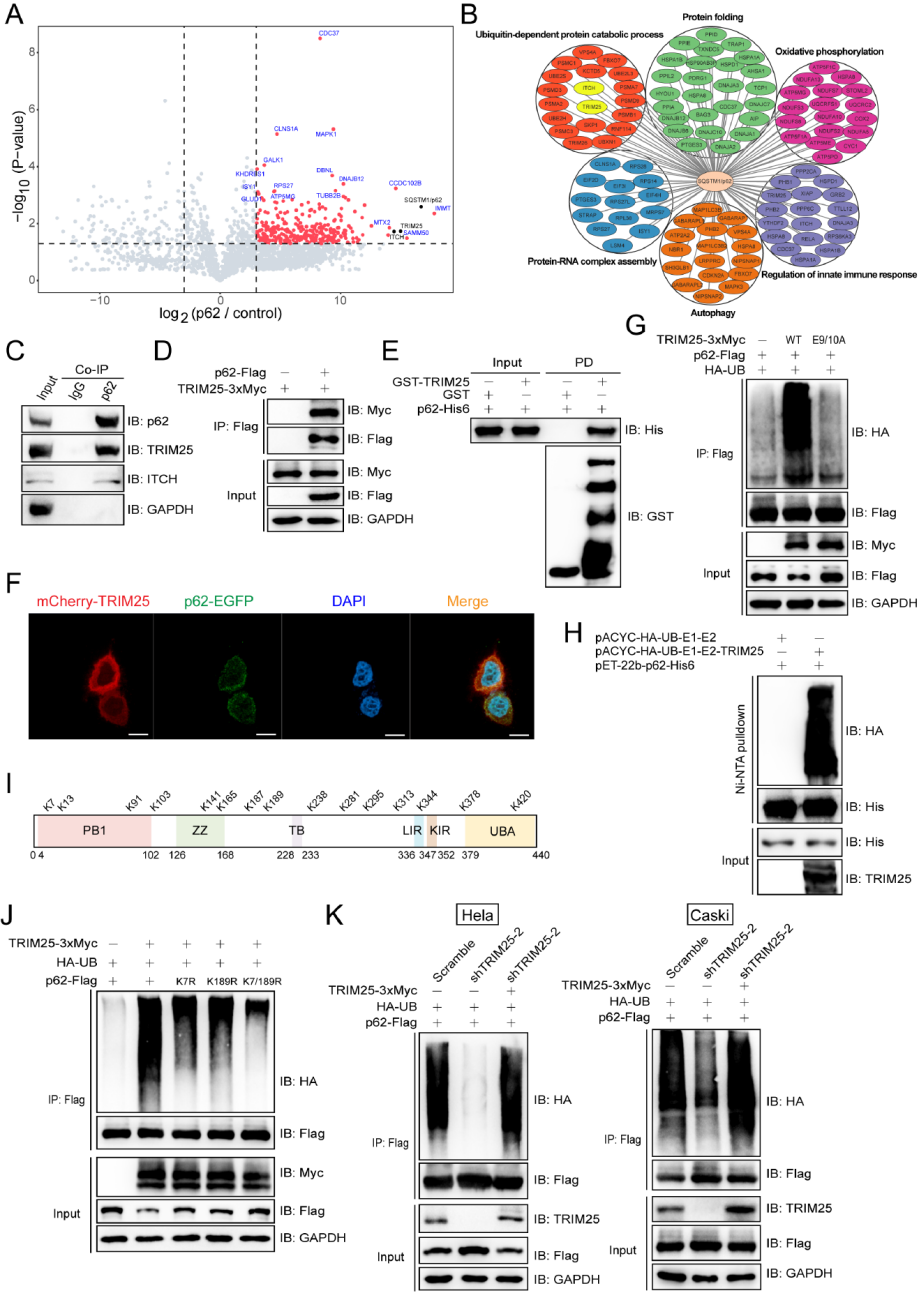
Figure 1 TRIM25 was identified as an E3 ubiquitin ligase for p62/SQSTM1 (A) Identification of p62-interacting proteins through co-immunoprecipitation followed by mass spectrometry analysis. HEK293T cells ectopically expressed Flag-tagged p62 or empty vectors for 48 h. Cell lysates were incubated with anti-Flag affinity gels, and co-immunoprecipitates were subjected to trypsin digestion followed by mass spectrometry analysis. Three samples per group. Volcano plots showing protein enrichment from co-immunoprecipitation experiments, with red circles representing proteins enriched more than 2-fold by p62 compared with empty vectors. (B) Gene Ontology (GO) analysis was performed on the p62-interacting proteins identified via mass spectrometry, and proteins enriched more than twofold in the p62 samples compared with the empty vector samples were selected for analysis. (C) Endogenous p62 formed a complex with the E3 ubiquitin ligases TRIM25 and ITCH. Endogenous p62 proteins from HEK293T cells were immunoprecipitated via anti-p62 antibodies, followed by immunoblotting with the indicated antibodies. (D) Ectopically expressed p62 formed a complex with TRIM25. Flag-tagged p62 or empty vectors were co-transfected with Myc-tagged TRIM25 co-expressed in HEK293T cells, which were subjected to a co-immunoprecipitation assay using an anti-Flag antibody, followed by immunoblotting analysis. (E) Recombinant TRIM25 directly interacted with p62. A GST pull-down assay was performed with recombinant Gst-tagged TRIM25 and His6-tagged p62. (F) Exogenously expressed p62 and TRIM25 were colocalized in HeLa cells. The plasmids encoding p62-EGFP and mCherry-TRIM25 were co-transfected into HeLa cells, and fluorescence was detected by microscopy. Scale bar: 10 μm. (G) Flag-tagged p62 was efficiently ubiquitinated by Myc-tagged TRIM25. HEK293T cells were co-transfected with the indicated plasmids, and cell lysates were immunoprecipitated using anti-Flag affinity gels, followed by immunoblotting analysis. TRIM25 (E9/10A) is an E3 ubiquitin ligase dead mutant. (H) TRIM25 ubiquitinated p62 in a reconstituted E. coli ubiquitination system. pACYC-E1-E2-TRIM25/PACYC-E1-E2 and pET22B-p62 were co-transformed into BL21 E. coli cells, and protein expression was induced by IPTG. The cell lysates were immunoprecipitated with Ni-NTA agarose beads and subjected to immunoblotting analysis. (I) Schematic distribution of the fifteen sites (Lys residues) involved in the TRIM25-mediated ubiquitination of p62. The p62 protein was recovered from the E. coli ubiquitination system (H) and subjected to mass spectrometry analysis to identify the ubiquitination sites. (J) TRIM25-mediated ubiquitination of p62 occurs mainly at two major ubiquitination sites (K7 and K189). Lysates from HEK293T cells ectopically expressing the indicated plasmids were immunoprecipitated using anti-Flag affinity gels, followed by immunoblotting analysis. (K) Reduced p62 ubiquitination by TRIM25 knockdown was reversed by exogenously expressed TRIM25. HeLa and Caski cells stably expressing TRIM25 shRNAs were co-transfected with the indicated plasmids, immunoprecipitated using anti-Flag affinity gels, and subjected to immunoblotting analysis.

Validation assays were then performed to test the E3 ubiquitin ligases for p62 identified in this study. Both endogenously and ectopically expressed p62 formed a complex with the E3 ubiquitin ligases TRIM25 and ITCH ( Figure 1C,D and Supplementary Figure S1A). GST pull-down assays revealed that recombinant TRIM25 and ITCH directly interact with p62 ( Figure 1E and Supplementary Figure S1B). As detected by fluorescence microscopy analysis, mCherry-tagged TRIM25 and GFP-tagged p62 were colocalized mainly in the cytoplasm of HeLa cells ( Figure 1F). Ubiquitination assays were performed to determine whether TRIM25 and ITCH are merely interacting partners or true E3 ubiquitin ligases for p62. One potential explanation for this phenomenon is that ITCH requires assistance from a specific protein or undergoes a particular modification to activate its ability to ubiquitinate p62. Alternatively, it is conceivable that p62 needs to be modified to be ubiquitinated by ITCH. Exogenously expressed p62 was efficiently ubiquitinated by TRIM25 but not by ITCH ( Figure 1G and Supplementary Figure S1C). These results suggest that TRIM25 is an E3 ubiquitin ligase for p62, whereas ITCH is only an interacting partner.

A reconstituted *E*. *coli* ubiquitination system, which has been used in several of our studies, was included in this study [ 5– 7]. The p62 proteins recovered from the *E*. *coli* ubiquitination system ( Figure 1H) were subjected to mass spectrometry analysis, and fifteen Lys (K) residues of p62 were identified ( Figure 1I). As shown in Supplementary Figure S1D, K7 and K189 were validated as the two major sites for the TRIM25-mediated ubiquitination of p62 in mammalian cells. The mutant bearing simultaneous K-to-R substitutions (K7/189R) at the two Lys residues almost completely abolished the TRIM25-mediated ubiquitination of p62 ( Figure 1J). Four shRNAs targeting *TRIM25* were designed and tested in HeLa and Caski cells ( Supplementary Figure S1E), and shTRIM25-1 and shTRIM25-2 were selected for further study. *TRIM25* knockdown reduced p62 ubiquitination ( Supplementary Figure S1F), which was effectively reversed by exogenously expressed TRIM25 in both HeLa and Caski cells ( Figure 1K).

In HEK293T cells, the TRIM25-mediated reduction in p62 protein expression was blocked by treatment with the autophagy inhibitor bafilomycin (BAF) but not by treatment with the proteasome inhibitor bortezomib (BTZ) ( Figure 2A). Upon undergoing ubiquitination, p62 enhances its interaction with LC3 through its LC3-interacting region (LIR) domain. This interaction enables p62 to be recruited to the phagophore membrane, where it facilitates the formation of autophagosomes. The subsequent fusion of the autophagosome with a lysosome leads to the degradation of both p62 and its associated ubiquitinated cargoes by lysosomal enzymes [8]. Treatment with cycloheximide (CHX), a protein synthesis inhibitor, led to TRIM25-mediated degradation of ectopically expressed p62 in HEK29T cells ( Figure 2B). Further analysis revealed that TRIM25 promoted the degradation of wild-type p62 but not the K7/189R mutant ( Figure 2C). The protein level of endogenous p62 was greater in *TRIM25*-knockdown cells than in control cells in the presence of CHX ( Figure 2D).

**Figure 2:**
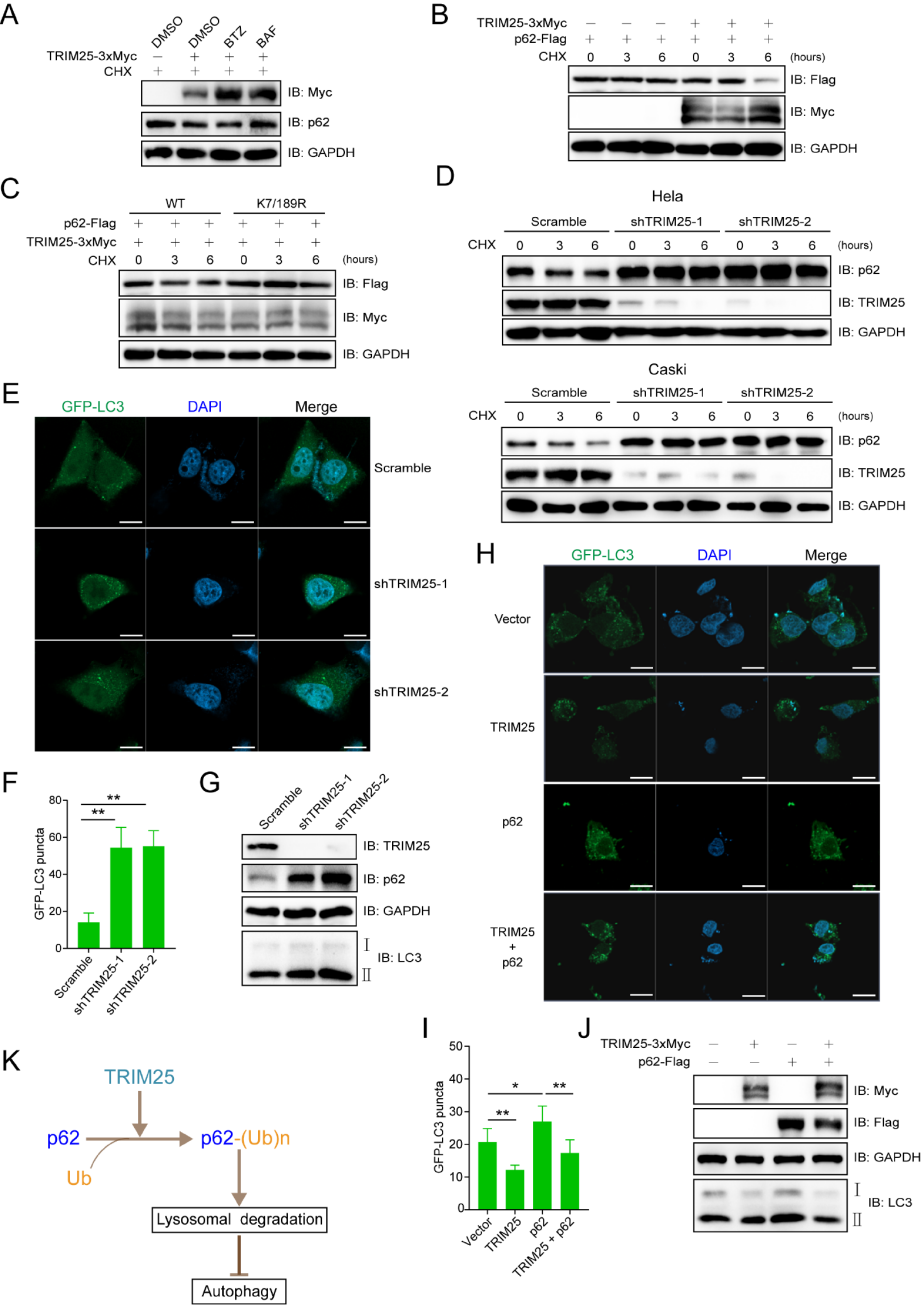
Figure 2 TRIM25 targets p62 for degradation and inhibits autophagy activation (A) TRIM25 mediated the degradation of p62 mainly through the lysosomal pathway. HeLa cells were treated with BTZ (1 μM) or BAF (20 nM) as well as CHX (100 μg/mL) for 6 h before harvesting. BTZ, bortezomib, a proteasome inhibitor; BAF, bafilomycin, an autophagy inhibitor; CHX, cycloheximide. (B) TRIM25 degraded p62 in the presence of CHX. HEK293T cells were transiently co-transfected with p62-Flag and TRIM25-Myc or empty vector and treated with CHX for different durations (0, 3 and 6 h) before immunoblotting analysis. (C) TRIM25 destabilized wild-type p62 but not the p62 (K7/189R) mutant. HEK293T cells were transiently cotransfected with plasmids encoding TRIM25-Myc and p62-Flag or p62 (K7/189R)-Flag and treated with cycloheximide for different durations before immunoblotting analysis. (D) TRIM25 knockdown stabilized p62. HeLa and Caski cells stably expressing TRIM25 shRNAs were treated with CHX for different durations before being subjected to immunoblotting analysis. (E,F) GFP-LC3 puncta formation was enhanced in TRIM25-knockdown cells. HeLa cells stably expressing TRIM25 shRNAs were transfected with GFP-LC3 for 48 h and subjected to fluorescence microscopy analysis (E). GFP-LC3 puncta were counted and quantified (F). **P < 0.01. Scale bar: 10 μm. (G) TRIM25 knockdown promoted autophagy. Lysates of HeLa cells stably expressing TRIM25 shRNAs were subjected to immunoblotting analysis. (H,I) TRIM25 inhibited p62-mediated GFP-LC3 puncta formation. HeLa cells were transfected with GFP-LC3 and the indicated plasmids for 48 h and subjected to fluorescence microscopy analysis (H). GFP-LC3 puncta were counted and quantified (I). *P < 0.05, **P < 0.01. Scale bar: 10 μm. (J) TRIM25 inhibited p62-mediated autophagy. HeLa cells were transfected with the indicated plasmids for 48 h and subjected to immunoblotting analysis. (K) Schematic of the TRIM25-p62 ubiquitination signaling pathway for autophagy regulation.

As p62 is an autophagy receptor, the effects of TRIM25-p62 ubiquitination signaling on the autophagy of cells were explored. GFP-LC3 puncta formation and LC3 II protein levels were greater in *TRIM25*-knockdown HeLa cells than in control cells ( Figure 2E–G). Further studies revealed that TRIM25 was able to reduce GFP-LC3 puncta formation and the level of the LC3 II protein activated by p62 ( Figure 2H–J).

Taken together, TRIM25 has been identified as a novel E3 ubiquitin ligase for p62. It targets p62 for lysosomal degradation, thereby inhibiting autophagy activation in mammalian cells ( Figure 2K). Our experiments were performed mainly in cell lines, so the relevance of TRIM25-p62 ubiquitination signaling in *in vivo* models remains unclear and needs to be further investigated. Although several proteins, including UBCH5A/B and TRIM21 [ 9, 10], have been identified as E3 ligases for p62, our study may provide new insights for the development of therapeutic strategies targeting these related pathophysiological processes.

## Supporting information

25230Supplementary_Data

## Supplementary Data

Supplementary data is available at *Acta Biochimica et Biophysica Sinica* online.

## References

[1] Klionsky DJ, Abdel-Aziz AK, Abdelfatah S, Abdellatif M, Abdoli A, Abel S, Abeliovich H (2021). Guidelines for the use and interpretation of assays for monitoring autophagy (4th edition). Autophagy.

[2] Cen X, Li Z, Chen X (2023). Ubiquitination in the regulation of autophagy. Acta Biochim Biophys Sin.

[3] Wang CC, Peng H, Wang Z, Yang J, Hu RG, Li CY, Geng WJ (2022). TRIM72-mediated degradation of the short form of p62/SQSTM1 rheostatically controls selective autophagy in human cells. Military Med Res.

[4] Liu H, Chai Z, Gao Y, Wang Y, Lu M (2024, 57: 995–1005). Ivermectin inhibits the growth of ESCC by activating the ATF4-mediated endoplasmic reticulum stress-autophagy pathway. Acta Biochim Biophys Sin.

[5] Li C, Han T, Guo R, Chen P, Peng C, Prag G, Hu R (2020). An integrative synthetic biology approach to interrogating cellular ubiquitin and ufm signaling. Int J Mol Sci.

[6] Xu X, Li C, Gao X, Xia K, Guo H, Li Y, Hao Z (2018). Excessive UBE3A dosage impairs retinoic acid signaling and synaptic plasticity in autism spectrum disorders. Cell Res.

[7] Yang Y, Luo Y, Yang C, Hu R, Qin X, Li C (2023). TRIM25-mediated ubiquitination of G3BP1 regulates the proliferation and migration of human neuroblastoma cells. Biochim Biophys Acta (BBA)-Gene Regulatory Mech.

[8] Kageyama S, Gudmundsson SR, Sou YS, Ichimura Y, Tamura N, Kazuno S, Ueno T (2021). p62/SQSTM1-droplet serves as a platform for autophagosome formation and anti-oxidative stress response. Nat Commun.

[9] Peng H, Yang J, Li G, You Q, Han W, Li T, Gao D (2017). Ubiquitylation of p62/sequestosome1 activates its autophagy receptor function and controls selective autophagy upon ubiquitin stress. Cell Res.

[10] Pan JA, Sun Y, Jiang YP, Bott AJ, Jaber N, Dou Z, Yang B (2016). TRIM21 ubiquitylates SQSTM1/p62 and suppresses protein sequestration to regulate redox homeostasis. Mol Cell.

