# Supplementary material for: TRIM25 ubiquitinates and degrades p62/SQSTM1 to suppress autophagy: TRIM25 ubiquitinates and degrades p62/SQSTM1
Source: Acta Biochim Biophys Sin (Shanghai). 2025 Jun 20;57(12):2129–32. doi: 10.3724/abbs.2025062 (PMC12747965; doi:10.3724/abbs.2025062)
Supplement: 25230Supplementary_Data [file 25230Supplementary_Data.docx]

**Supplementary Materials and Methods**

**Plasmid construction**

Plasmids containing TRIM25, p62 and ITCH were generated from human cDNA from HEK293T cells, and then the amplified full-length or fragmentary cDNA was inserted into vectors such as PACYC, pCADNA3.0-Flag/MYC, pET22b, or pGEX4T-1. Point mutations of the indicated plasmids containing TRIM25 or p62 were introduced via site-directed mutagenesis. The TRIM25 shRNAs used in this study were previously described [1].

**Cell culture and transfection**

Human embryonic kidney 293T (HEK293T) and cervical cancer HeLa and Caski cell lines were obtained from the Cell Bank of the Chinese Academy of Sciences (Shanghai, China) and cultured in high-glucose DMEM supplemented with 10% fetal bovine serum (FBS; Gibco, Carlsbad, USA), 100 mg/mL streptomycin and 100 U/mL penicillin (Gibco) in a humidified incubator with 5% CO_2_ at 37°C. The plasmids were transfected into the cells via Lipofectamine 2000 (Life Technologies, Carlsbad, USA) according to the manufacturer’s instructions.

**Identification of p62-interacting proteins via mass spectrometry**

Six 10-cm dishes of HEK293T cells were transfected with empty vectors or pCDNA3.0-p62-Flag via Lipofectamine 2000 (Invitrogen, Carlsbad, USA) and cultured for 48 h. The cells were then lysed in Co-IP buffer (50 mM Tris-HCl, 150 mM NaCl, 5 mM EDTA and 1% NP-40 pH 7.6) supplemented with protease inhibitor cocktail and incubated with anti-Flag affinity gels at 4°C overnight. The immunoprecipitates were washed three times with Co-IP buffer and eluted with 8 M urea dissolved in 100 mM Tris-HCl (pH 8.0), followed by TCEP reduction, CAA alkylation and trypsin digestion, and subjected to mass spectrometry as previously described [2]. The raw MS files were analysed as previously described [2], and the differentially expressed proteins (DEPs) and proteins detected (intensity > 0) in at least three samples were considered. Missing values were imputed with the minimum value across our proteome data. The data were subsequently normalized on the basis of quantiles. A one-sided *t* test (as implemented in R software) was used to assess the DEPs. The P value was adjusted via the Benjamini & Hochberg method. Proteins with adjusted P values ≤0.05 and fold changes ≥ 2 were considered upregulated proteins. Gene Ontology (GO) enrichment analysis was performed with Metascape (https://Metascape.org/). An adjusted P value ≤0.05 was considered statistically significant. The network was visualized via Cytoscape. The mass spectrometry proteomics data have been deposited in the ProteomeXchange Consortium via the PRIDE partner repository with the dataset identifier PXD061794.

**Co-immunoprecipitation (Co-IP), immunoprecipitation and immunoblotting**

For the Co-IP assay, cells transfected with the indicated plasmids were lysed in 1 ml of Co-IP buffer supplemented with a protease inhibitor cocktail (Roche, Basel, Switzerland), centrifuged and incubated overnight at 4°C with anti-Flag affinity gels (Sigma, St Louis, USA) or an anti-p62 antibody (1:100 dilution, 66184-1-Ig, Proteintech, Wuhan, China) plus protein G magnetic beads (L-1002, Biolinkedin, Shanghai, China). For the immunoprecipitation (IP) assay, the cells were lysed in 500 μL of RIPA buffer (150 mM NaCl, 50 mM Tris-HCl, 5 mM EDTA, 1% NP-40 and 0.1% SDS, pH 7.8) containing freshly added protease inhibitor cocktail. Then, the cell lysates were incubated with anti-Flag affinity gels at 4°C. The next day, the immunoprecipitates were washed three times with Co-IP/IP buffer before denaturation at 100°C for 10 min in 2× SDS protein loading buffer. The immunoprecipitates and inputs were subjected to 10% or 15% SDS-PAGE and transferred to PVDF (polyvinylidene fluoride) membranes (Bio-Rad, Hercules, USA). The membranes were blocked with 10% skim milk at room temperature (RT) for 1 h and then incubated with the following specific antibodies: anti-TRIM25 (1:1000 dilution; 12573-1-AP; Proteintech), anti-p62 (1:1000 dilution; 18420-1-AP, Proteintech), anti-ITCH (1:1000 dilution; 20920-1-AP; Proteintech, China), anti-GAPDH (1:8000 dilution; 60004-1-Ig; Proteintech), anti-Flag (1:2000 dilution; 80010-1-RR; Proteintech), anti-MYC (1:10000 dilution; 16286-1-AP; Proteintech), anti-HA (1:5000 dilution; 51064-2-AP; Proteintech), anti-GST (1:5000 dilution; HRP-66001; Proteintech) or anti-His (1:1000 dilution; 66005-1-Ig; Proteintech). The membranes were then incubated with the following horseradish peroxidase-labelled secondary antibodies: goat anti-rabbit IgG (1:5000 dilution; SA00001-2; Proteintech) or goat anti-mouse IgG (1:5000 dilution; SA00001-1; Proteintech). The signals were detected via a Tanon 5200 imaging system (Tanon, Beijing, China).

**Recombinant protein purification**

GST- and His6-tagged proteins were expressed in the BL21 *E. coli* system. Protein expression was induced with isopropyl-β-d-mercapto-galactopyranoside (IPTG; Sagon, Shanghai, China). The cells were then centrifuged, lysed in PBS before incubation with glutathione or Ni-NTA agarose beads and eluted with 20 mM reduced L-glutathione solution or 500 mM imidazole. The eluate was dialyzed overnight at 4°C in PBS buffer containing 20% glycerol and stored at −80°C.

**GST pull-down assay**

Purified His6-tagged proteins (10 μg), GST-tagged proteins (10 μg), and glutathione Sepharose4B (Sangon) were incubated overnight at 4°C in 1 mL of GST pull-down buffer (20 mM Tris-Cl, 100 mM NaCl, 5 mM MgCl_2_, 1 mM EDTA, 0.5% NP-40 and fresh 1 mM DTT, pH 8.0) supplemented with fresh 10 mg/mL BSA. The samples were pelleted and washed three times with GST pull-down buffer as described above. The immunoprecipitates were then boiled in 40 μL of 2x SDS protein loading buffer for 10 minutes before being subjected to immunoblotting analysis.

**Reconstituting the *E. coli* ubiquitination system and mapping p62 ubiquitination sites**

E1 (UBA1), E2 (UBCH13) and HA-UB were added to the first multiple cloning site (MCS) of the PACYC vector, and TRIM25 was added to the second multiple cloning site to construct the plasmid pACYC-HA-UB-UBCH13-UBA1-TRIM25, which was cotransformed with pET22B-p62-His6 in BL21 *E. coli* competent cells and screened with ampicillin plus chloramphenicol antibiotics. After induction with IPTG, the cells were lysed with 8 M urea lysis buffer (50 mM Tris-Cl, 50 mM Na_2_HPO_4_, 300 mM NaCl, 8 M urea, 0.5% NP-40 and 20 mM imidazole, pH 8.0) and then incubated with Ni-NTA agarose beads for 4 hours at room temperature. The immunoprecipitates were washed three times with 8 M urea lysis buffer and denatured in 2× SDS protein loading buffer at 100°C for 10 min before immunoblotting analysis. The p62 proteins recovered from the *E. coli* ubiquitination system were subjected to mass spectrometry analysis for ubiquitination site mapping as previously described [3]. The sample was dissolved in 8 M urea and 100 mM Tris-Cl (pH 8.5), followed by TCEP reduction, NEM alkylation and trypsin digestion. Peptides were separated via the EASY-nLC system (Thermo Fisher Scientific, Waltham, USA) and analysed via a Q Exactive mass spectrometer (Thermo Fisher Scientific). Protein and ubiquitination analyses were performed via Thermo Proteome Discoverer 2.1 (Thermo Fisher Scientific), and the results were compared against those of the UniProt Human database (http://www.uniprot.org/). The mass spectrometry data have been deposited to the ProteomeXchange Consortium via the PRIDE partner repository with the dataset identifier PXD062511.

**Fluorescence microscopy analysis**

For colocalization analysis, HeLa cells were transfected with plasmids encoding mCherry-tagged TRIM25 and GFP-tagged p62 for 24 h, fixed with 4.0% PFA (paraformaldehyde; Sigma, St Louis, USA) and stained with DAPI (4',6-diamidino-2-phenylindole; Sigma) as described previously[2]. Images were captured via a BX51 microscope (Olympus, Tokyo, Japan). For GFP-LC3 puncta formation analysis, HeLa cells were transfected with plasmids encoding GFP-LC3 for 48 h. The cells were then fixed with 4% PFA for 15 min, and the nuclei were counterstained with DAPI. The fluorescence was detected via a BX51 microscope. GFP-LC3 puncta formation was quantified by counting and calculating the number of GFP-LC3 puncta in ten cells from six fields.

**Statistical analysis**

The data generated in this study are expressed as the mean ± standard deviation (SD) and were analysed via GraphPad Prism 7 (GraphPad Software Inc., San Diego, USA). Statistical significance was determined via a two-tailed unpaired t test or one-way ANOVA with Tukey’s post hoc test. A *P* value of less than 0.05 was considered to indicate a significant difference, whereas a P value of less than 0.01 was considered to indicate a very significant difference.

**References**

1. Yang Y, Luo Y, Yang C, Hu R, Qin X, Li C. TRIM25-mediated ubiquitination of G3BP1 regulates the proliferation and migration of human neuroblastoma cells. Biochim Biophys Acta Gene Regul Mech 2023, 1866: 194954

2. Li C, Han T, Li Q, Zhang M, Guo R, Yang Y, Lu W*, et al.* MKRN3-mediated ubiquitination of Poly(A)-binding proteins modulates the stability and translation of GNRH1 mRNA in mammalian puberty. Nucleic Acids Res 2021, 49: 3796-3813

3. Li C, Han T, Guo R, Chen P, Peng C, Prag G, Hu R. An Integrative Synthetic Biology Approach to Interrogating Cellular Ubiquitin and Ufm Signalling. Int J Mol Sci 2020, 21

**Supplementary Table S1. SQSTM1/p62-interacting proteins identified by mass spectrometry analysis**


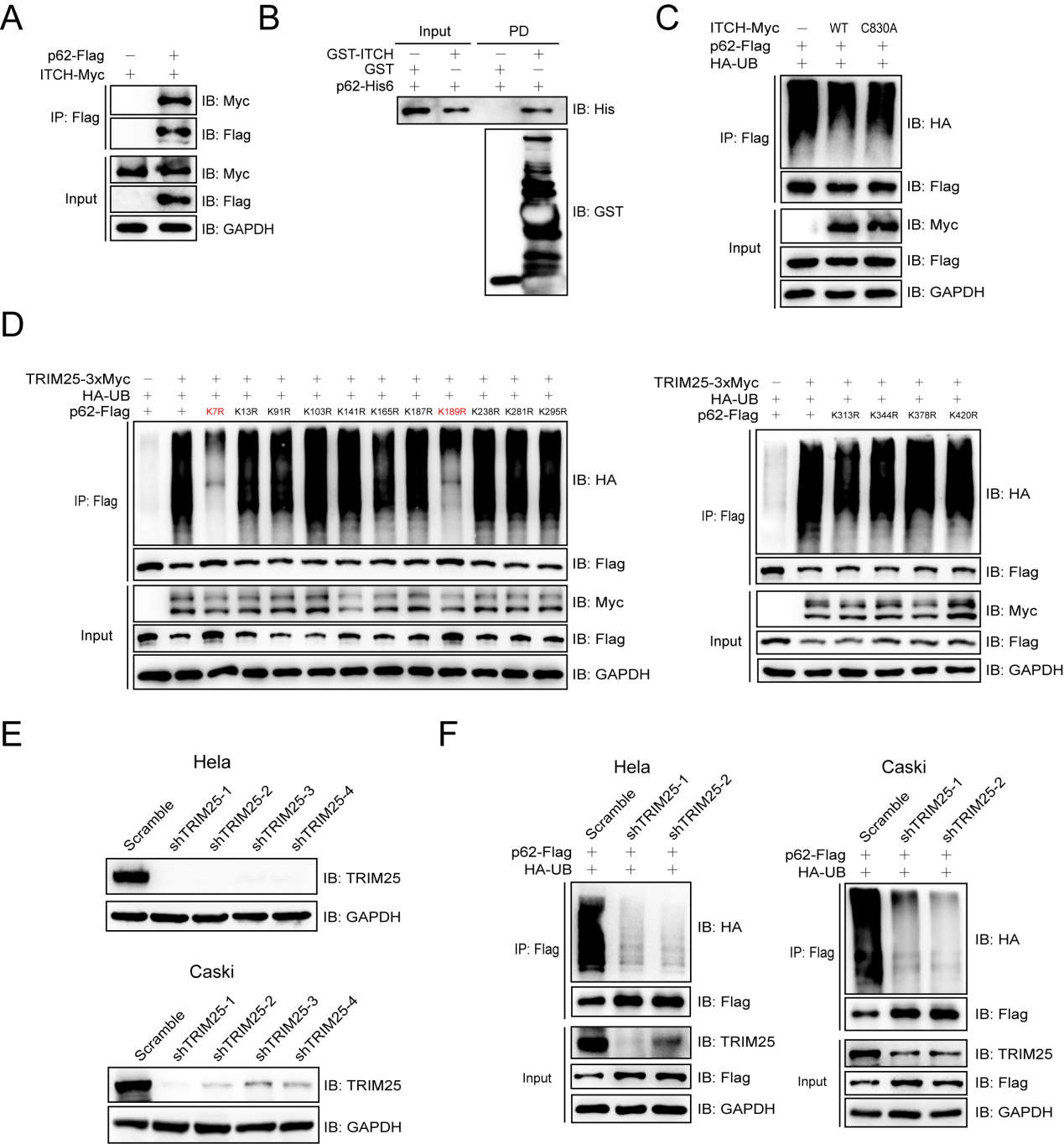


**Supplementary Figure S1. TRIM25 was identified as an E3 ubiquitin ligase for p62/SQSTM1** (A) Ectopically expressed p62 formed a complex with ITCH. Flag-tagged p62 or empty vectors were co-transfected with Myc-tagged ITCH co-expressed in HEK293T cells, which were subjected to co-immunoprecipitation assay using an anti-Flag antibody, followed by immunoblotting analysis. (B) Recombinant ITCH directly interacted with p62. A GST pull-down assay was performed with recombinant Gst-tagged ITCH and His6-tagged p62. (C) Myc-tagged ITCH was not capable of facilitating the ubiquitination of Flag-tagged p62. HEK293T cells were co-transfected with the indicated plasmids, and cell lysates were immunoprecipitated using anti-Flag affinity gels, followed by immunoblotting analysis. ITCH (C830A) is a dead mutant of the E3 ubiquitin ligase. (D) Two Lys (K) residues, K7 and K189 (shown in red), were shown to be the major sites for the TRIM25-mediated ubiquitination of p62. Lysates of HEK293T cells ectopically expressing HA-Ub, TRIM25-Myc, p62-Flag or the indicated K-to-R mutants were immunoprecipitated with anti-Flag affinity gels, followed by immunoblotting analysis. (E) Test of the knockdown efficiency of shRNAs targeting TRIM25. shRNAs for TRIM25 were transfected into HeLa and Caski cells, and stably expressing cell lines were established via puromycin selection. The protein levels of TRIM25 were detected by immunoblotting analysis. (F) TRIM25 knockdown reduced the ubiquitination of p62. HeLa and Caski cells stably expressing TRIM25 shRNAs were co-transfected with the indicated plasmids, immunoprecipitated using anti-Flag affinity gels, and subjected to immunoblotting analysis.
